# The impact of long-term organic farming on soil-derived greenhouse gas emissions

**DOI:** 10.1038/s41598-018-38207-w

**Published:** 2019-02-08

**Authors:** Colin Skinner, Andreas Gattinger, Maike Krauss, Hans-Martin Krause, Jochen Mayer, Marcel G. A. van der Heijden, Paul Mäder

**Affiliations:** 1 Department of Soil Sciences, Research Institute of Organic Agriculture (FiBL), Ackerstrasse, CH-5070 Frick, Switzerland; 2 Organic Farming with focus on Sustainable Soil Use, Institute of Crop Science and Breeding II, Justus-Liebig University Giessen, 35394 Giessen, Germany; 3 Water Protection and Substance Flows, Research Division Agroecology and Environment, Agroscope, CH 8046 Zürich, Switzerland; 4 Plant Soil Interactions, Research Division Agroecology and Environment, Agroscope, CH 8046 Zürich, Switzerland; 5 Department of Evolutionary Biology and Environmental Studies, University of Zürich, CH 8057 Zürich, Switzerland; 6 Institute of Environmental Biology, Faculty of Science, Utrecht University, 3508 TC Utrecht, The Netherlands

## Abstract

Agricultural practices contribute considerably to emissions of greenhouse gases. So far, knowledge on the impact of organic compared to non-organic farming on soil-derived nitrous oxide (N_2_O) and methane (CH_4_) emissions is limited. We investigated N_2_O and CH_4_ fluxes with manual chambers during 571 days in a grass-clover– silage maize – green manure cropping sequence in the long-term field trial “DOK” in Switzerland. We compared two organic farming systems – biodynamic (BIODYN) and bioorganic (BIOORG) – with two non-organic systems – solely mineral fertilisation (CONMIN) and mixed farming including farmyard manure (CONFYM) – all reflecting Swiss farming practices–together with an unfertilised control (NOFERT). We observed a 40.2% reduction of N_2_O emissions per hectare for organic compared to non-organic systems. In contrast to current knowledge, yield-scaled cumulated N_2_O emissions under silage maize were similar between organic and non-organic systems. Cumulated on area scale we recorded under silage maize a modest CH_4_ uptake for BIODYN and CONMIN and high CH_4_ emissions for CONFYM. We found that, in addition to N input, quality properties such as pH, soil organic carbon and microbial biomass significantly affected N_2_O emissions. This study showed that organic farming systems can be a viable measure contributing to greenhouse gas mitigation in the agricultural sector.

## Introduction

With a share of 10–12% in carbon-dioxide equivalents (CO_2_-eq.), agriculture contributes substantially to global greenhouse gas (GHG) emissions^1^. Considering indirect emissions from agriculture-related activities such as fertiliser production and land use change, this share can be up to 30%^2^. In 2005, the agricultural sector contributed 56% to the emissions of the anthropogenic non-CO_2_, GHG, i.e. nitrous oxide and methane, with an annual growth of 0.9%^1^. An estimated 38% of agriculture’s direct emissions originate from soils, 15% from N_2_O from manure on pasture and 12% from N_2_O of synthetic fertilisers – the latter at an annual growth rate of 3.9% from 1961 to 2010. In comparison, paddy rice cultivation contributes 11% to the sector’s annual emissions, mainly in the form of CH_4_ ^1^. Due to their high global warming potential (GWP) the reduction of N_2_O and CH_4_ emissions thus gives agriculture’s mitigation potential regarding climate change a high leverage in favour of limiting Global Warming^3^.

Fostered by the Green Revolution and the availability of cheap synthetic fertilisers from the 1950s onward, world grain harvests doubled to 2.5 billion tons between 1970 and 2010 with an average yield increase from 1600 to 3030 kg ha^−1^ ^1^. However, this agricultural intensification was realized mainly through the increase in global fertiliser usage from 32 to 106 Mt yr^−1^ (+331%). Moreover, these achievements have been accomplished at the expense of environmental damages such as biodiversity loss, accelerated soil erosion and degradation, eutrophication including algal blooms and oceanic dead zones, or pesticide effects on humans and wildlife^4^. There is an increased understanding that the challenges of producing enough food and biomass while maintaining ecosystem services cannot be met by modern intensive “non-organic” agricultural practices that rely heavily on synthetic fertiliser input and pesticide application^4^. Thus, agro-ecological approaches towards an ecological intensification are fundamental for future food production^5^. Certified “organic” farming is considered as one option towards ecological intensification^5^. Reganold and Wachter recently examined the performance of organic farming in the light of the four key sustainability metrics: productivity, environmental impact, economic viability and social wellbeing. They concluded that, despite lower yields, organic farming delivers greater ecosystem services and social benefits. Thus, organic farming practices need to be considered for the development of sustainable farming systems, which presumably will encompass a blend of organic together with non-organic approaches^6^.

However, there are still knowledge gaps regarding the effects of organic farming on soil carbon (C) and nitrogen (N) fluxes, and thus on N_2_O and CH_4_ greenhouse gas emissions from the soil^7^. Organic fertilisation strategies through farmyard manure and cropping of N-fixing legumes, grass-clover leys and catch crops influence soil C and N fluxes in complex processes. These can run over years and decouple fertiliser inputs and availability of nutrients^8^. Investigating these processes requires long-term measurements, at best covering complete crop rotations. Skinner *et al*. performed a literature search on measured soil-derived nitrous-oxide and methane fluxes under organic and non-organic management from comparison trials of farming systems, followed by a meta-analysis of the aggregated global dataset. Only 19 studies based on field measurements could be retrieved, of which 13 covered annual measurements. Based on 12 studies comprising annual measurements within the land-use types arable and grassland, lower area-scaled N_2_O emissions and (three studies) higher CH_4_ uptake in organic compared to non-organic farming systems were determined^8^. However, related to crop yield (eight studies), organically managed soils emitted more N_2_O than soils in non-organic systems due to the yield gap of 26% between organic and non-organic farming. Reviews of global datasets resulted with a gap of around 20%^8–10^. Yet, due to the small number of studies and the average duration of contrasting management of only nine years, further evidence originating from long-term experiments is needed. Along this line, Van Kessel *et al*. found in their global meta-study that management-induced impacts on the N_2_O emission pattern become effective only after more than 10 years since adoption of reduced tillage practices^11^.

There are different kinds of organic farming systems unified under the umbrella of the International Federation of Organic Agricultural Movements IFOAM (www.ifoam.bio)^12^. Among these, biodynamic agriculture as proposed by Steiner in 1924 is the oldest certified organic farming type^13,14^. Even though considerable parts of Steiner’s biodynamic philosophy and practices lie beyond scientific judgement, a fair share of the available peer-reviewed research results from controlled field experiments as well as case studies show effects of biodynamic farming on yield, soil quality and soil biodiversity^15–19^. However, so far it has not been tested whether the effects of biodynamic farming on GHG emissions differ from other organic farming systems.

The DOK long-term field trial established in 1978 compares organic and non-organic farming systems^15^. The differentiated management of the farming systems compared since the start has led to system-specific changes of soil physical, chemical and biological properties^15,18,19^. By measuring soil gas fluxes in the farming systems of the DOK trial, we aim to extend the knowledge base concerning the impact of organic farming on direct GHG emissions. Two organic farming systems – bioorganic (BIOORG; with slurry and rotted manure) and biodynamic (BIODYN; with slurry and composted manure) - and two non-organic – conventional with livestock (CONFYM; with slurry, fresh stacked manure and synthetic fertilisers) and without (CONMIN; only synthetic fertilisers), all fertilised to an intensity typically practiced in Switzerland were chosen for the experiment. We included the unfertilised control, NOFERT, as a reference. All farming systems are subject to the same crop rotation, but plant protection in the organic and the unfertilised system is based on mechanical weeding, indirect disease control measures and plant extracts together with bio-controls against insects, while in the non-organic systems herbicides, fungicides and pesticides are applied. The monitoring covered a cropping sequence consisting of grass-clover in the second year followed by silage maize (cv. “Colisee”) and green manure (Brassica chinensis x Brassica rapa) covering 571 days with 86 samplings.

Based on the findings of aforesaid study^8^, which serves as a topical review to present field research, we hypothesised that (i) area-scaled N_2_O emissions are lower, (ii) yield-scaled N_2_O emissions are higher and (iii) CH_4_ uptake is higher in organic compared to non-organic farming systems. Furthermore, we aimed at investigating possible effects of biodynamic farming and identifying major drivers for the observed findings to suggest mitigation options.

## Results

### N_2_O and CH_4_ flux dynamics

**Figure 1:**
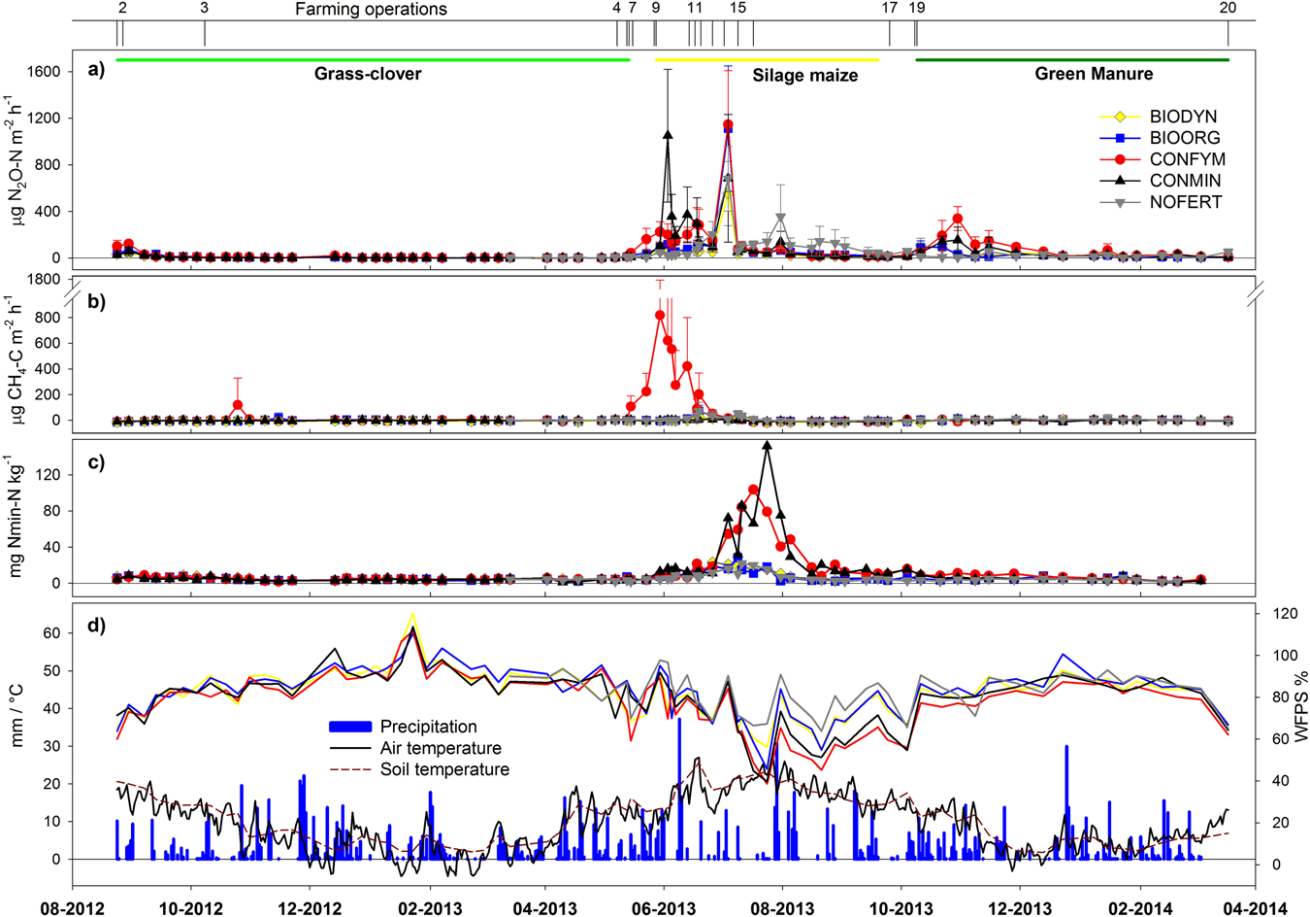
Temporal dynamics of N_2_O-N (**a**) and CH_4_-C (**b**) fluxes during the grass-clover – silage maize – green manure cropping sequence (24 Aug 2012 to 18 Mar 2014) in the different farming systems of the DOK long-term field trial. Numbers on top of the panels indicate management operations; detailed information is given in supplementary table S1. The horizontal coloured lines in panel (a) indicate cropping seasons, gaps are fallows. Panel (c) depicts the temporal dynamics of N_min_ contents (0–20 cm) per system. Panel (d) shows the temporal dynamics of water filled pore space (WFPS) with the scale on the right side (line colours indicate the systems) and precipitation, air- and soil temperature (10 cm depth) with the scale on the left-hand side.

For all investigated farming systems N_2_O fluxes were lowest during the grass-clover phase and highest during the silage maize period with two major emission events (Fig. 1). The first one consisted of distinctly raised fluxes in CONFYM after solid manure application and ploughing, together with peak fluxes in CONMIN that immediately followed the initial mineral fertilisation and sowing of silage maize. The second N_2_O emission pulse, pronounced in all systems, developed after the second fertiliser application during silage maize cultivation (Fig. 1; Supplementary Table S1). Enhanced N_2_O fluxes at the beginning of the green manure phase that followed silage maize harvest amount to between 20 and 34% of total cumulated area scale N_2_O emissions for the entire crop sequence, although no additional N was applied (Fig. 1; Table 1).

**Table 1:** Means and standard errors (SE) of cumulative area-scaled N_2_O emissions (kg N_2_O-N ha^−1^) of a grass-clover – silage maize – green manure sequence.

Farming System	Grass-clover (24 Aug 2012 to 7 May 2013, 256 days)		Silage Maize (7 May 2013 to 24 Sep 2013, 140 days)		Green Manure (24 Sep 2013 to 18 Mar 2014, 175 days)		Overall (24 Aug 2012 to 18 Mar 2014, 571 days)		Annual data (18 Mar 2013 to 18 Mar 2014)	
	Mean	SE	Mean	SE	Mean	SE	Mean	SE	Mean	SE
BIODYN	0.30	0.04bc	2.07	0.38b	1.15	0.29bc	3.67	0.40c	3.38	0.38b
BIOORG	0.40	0.03ab	3.55	0.23ab	0.89	0.06bc	5.04	0.34bc	4.66	0.33b
CONFYM	0.49	0.02a	4.75	0.49a	2.81	0.51a	8.16	0.47a	7.68	0.48a
CONMIN	0.26	0.04c	4.22	0.85a	1.59	0.36ab	6.16	0.94ab	5.91	0.93ab
NOFERT	—^a^		4.17	1.03a	0.73	0.17c	—^a^		5.03	1.08ab
**ANCOVA**	**F-value**	**p-value**	**F-value**	**p-value**	**F-value**	**p-value**	**F-value**	**p-value**	**F-value**	**p-value**
(Intercept)	204.44	<0.0001	155.84	<0.0001	4.37	0.006	248.13	<0.0001	732.44	<0.0001
Clay content	16.04	0.003	1.10	0.316	9.98	0.009	1.62	0.238	4.00	0.070
Farming System	9.28^a^	0.005	3.38	0.049	7.70	0.003	10.68^a^	0.003	4.56	0.020
**Contrasts**	**t-value**	**p-value**	**t-value**	**p-value**	**t-value**	**p-value**	**t-value**	**p-value**	**t-value**	**p-value**
org vs. non-org	−0.96	0.333	−2.61	0.008	−4.31	<0.0001	−4.94	<0.0001	−3.95	<0.0001

^a^Measurements in NOFERT started at 14 March 2013 and thus were excluded from statistical analysis.

ANCOVA on log scaled N_2_O emissions per farming system (n = 4). Letters = Post-hoc Tukey test (p < 0.05).

Contrasts: Post-hoc t-test on pairwise comparisons of org (BIODYN + BIOORG) and non-org (CONFYM + CONMIN) systems (n = 8).

Overall, N_2_O flux dynamics were best explained by soil moisture expressed as water-filled pore space (WFPS) (p < 0.0001), followed by soil nitrate concentrations (p = 0.021). Freeze-thaw induced N_2_O fluxes were not observed during winter periods 2012/2013 and 2013/2014. The prerequisite condition for frost/thaw events, frozen soil, did not occur (Fig. 1, Supplementary Fig. S1).

CH_4_ emissions differed between farming systems with slight CH_4_ uptake in BIODYN and CONMIN and no or low CH_4_ net emissions in BIOORG and NOFERT (Table 2). CONFYM was a strong source for CH_4_, with peaking fluxes starting immediately after solid manure application and ploughing and lasting until the second fertilisation of the silage maize. Weather conditions during this period were rather cool with excessive rainfall (Fig. 1; Supplementary Fig. S1; Table 2).

**Table 2:** Means and standard errors (SE) of cumulative CH_4_ emissions (kg CH_4_-C ha^−1^) of a grass-clover – silage maize – green manure sequence.

Farming System	Grass-clover (24 Aug 2012 to 7 May 2013, 256 days)		Silage Maize (7 May 2013 to 24 Sep 2013, 140 days)		Green Manure (24 Sep 2013 to 18 Mar 2014, 175 days)		Overall (24 Aug 2012 to 18 Mar 2014, 571 days)		Annual data (18 Mar 2013 to 18 Mar 2014)	
	Mean	SE	Mean	SE	Mean	SE	Mean	SE	Mean	SE
BIODYN	−0.27	0.02b	−0.17	0.04b	0.00	0.03a	−0.48	0.04b	−0.26	0.05b
BIOORG	−0.13	0.06ab	0.02	0.07ab	0.03	0.06a	−0.09	0.15ab	0.00	0.12ab
CONFYM	0.05	0.14a	3.31	1.26a	−0.03	0.04a	3.29	1.21a	3.21	1.31a
CONMIN	−0.29	0.14ab	−0.13	0.03b	−0.06	0.06a	−0.49	0.20ab	−0.26	0.11b
NOFERT	—^a^		0.19	0.14ab	0.15	0.04a	—^a^		0.33	0.15ab
**Kruskal Wallis test**	**χ** ^**2**^	**p-value**	**χ** ^**2**^	**p-value**	**χ** ^**2**^	**p-value**	**χ** ^**2**^	**p-value**	**χ** ^**2**^	**p-value**
Farming System	7.70^a^	0.053	14.56	0.006	8.31	0.081	10.48^a^	0.015	14.64	0.006
org vs. non-org	1.86	0.172	2.16	0.141	0.89	0.345	2.48	0.115	1.59	0.208

^a^Measurements in NOFERT started at 14 March 2013 and thus were excluded from statistical analysis.

Kruskal Wallis non-parametric test on CH_4_ emissions per farming system (n = 4) and pairwise comparisons of org (BIODYN + BIOORG) and non-org (CONFYM + CONMIN) systems (n = 8). Letters = Post-hoc Kruskal Nemeny test (p < 0.05).

### Area-scaled GHG emissions

Cumulated over the whole observation period of 571 days mean N_2_O emissions ranged from 3.67 (BIODYN) to 8.16 kg N_2_O-N ha^−1^ (CONFYM) (Table 1). Related to a one-year period, with GHG flux data being recorded from all five farming system treatments (including NOFERT), annual N_2_O emissions varied between 3.38 (BIODYN) to 7.68 kg N_2_O-N ha^−1^ (CONFYM). This annual dataset encompasses samplings in silage maize and green manure. For all observation periods a significant impact of farming systems on N_2_O emissions was observed (p = 0.005; 0.049; 0.003; 0.003 and 0.020) (Table 1). Except for the grass-clover phase, we detected significantly lower area-scaled N_2_O emissions for the cropping periods “silage maize” and “green manure” between organic (BIODYN, BIOORG) compared to the non-organic (CONFYM, CONMIN) systems, as revealed by contrast analysis (p = 0.008; <0.0001). For the silage-maize cropping period lasting 140 days we found lowest N_2_O emissions with 2.07 kg N_2_O-N ha^−1^ in BIODYN and highest in CONFYM (4.75 kg N_2_O-N ha^−1^). BIODYN showed significantly (p < 0.05) lowest N_2_O emissions for the overall and silage maize observation periods. N_2_O emissions for the unfertilised treatment NOFERT under silage maize nearly matched those from fertilised non-organic treatments.

Cumulated over the whole observation period of 571 days, CH_4_ emissions ranged from −0.49 (CONMIN) to +3.29 kg CH_4_-C ha^−1^ (CONFYM) (Table 2). Statistical analyses revealed a farming systems effect with significantly (p = 0.015) highest CH_4_ emissions in CONFYM but no differences between both organic and non-organic systems. The effect of farming systems was most pronounced (p = 0.006) during silage maize cropping (Table 2).

### Yield-scaled N_2_O emissions

Yield-scaled N_2_O emissions were determined only for silage maize. We recorded by far the lowest yield-scaled N_2_O emissions in BIODYN (Table 3). The highest yield-scaled N_2_O emissions for silage-maize were observed in NOFERT with 478.8 g N_2_O-N Mg^−1^ dry matter (Table 3). Yield-scaled N_2_O emissions in BIOORG, CONFYM and CONMIN were on a medium level in between. Maize yields in 2013 were highest in the non-organic systems CONFYM/CONMIN and significantly (p < 0.0001) lower in the organic systems BIODYN/BIOORG. NOFERT achieved about half of the yield of CONFYM (Table 3). The organic yield gap, calculated on the averages of both organic vs. both non-organic farming systems, amounted to 27% in 2013.

**Table 3:** Silage maize yields and yield-scaled N_2_O emissions (mean ± SE).

	Silage maize DM^a^ yields			Yield-scaled N_2_O emissions	
	Mg DM ha^−1^		% of CONFYM	g N_2_O-N Mg DM^−1^	
**Farming system effect**					
BIODYN	13.57 ± 0.70b		73	154.8 ± 31.0b	
BIOORG	13.21 ± 0.23b		71	269.0 ± 17.5ab	
CONFYM	18.62 ± 1.22a		100	259.1 ± 32.7ab	
CONMIN	18.00 ± 0.77a		97	238.3 ± 52.5ab	
NOFERT	8.65 ± 0.35c		47	478.8 ± 113.9a	
**ANCOVA**	**F-value**	**p-value**		**F-value**	**p-value**
(Intercept)	10577.4	<0.0001		3489.5	<0.0001
Clay content	7.06	0.022		0.21	0.652
Farming System	33.53	<0.0001		3.94	0.031
**Contrasts**	**t-value**	**p-value**		**t-value**	**p-value**
org vs. non-org	−7.17	<0.0001		−0.59	0.550

^a^Dry matter (DM) 80%

ANCOVA per farming system (n = 4). Letters = Post-hoc Tukey test (p < 0.05).

Contrasts: Post-hoc t-test on pairwise comparisons of org (BIODYN + BIOORG) and non-org (CONFYM + CONMIN) systems (n = 8).

### Drivers of cumulative N_2_O emissions

To identify the impact of N inputs and soil biogeochemical parameters on N_2_O emissions, we compared N_2_O emissions of silage maize in the 2013 cropping period with N input and the soil parameters, pH, soil organic carbon (SOC) and microbial biomass carbon (C_mic_) as determined 2012 in the grass-clover ley (Supplementary Table S2). Since the unfertilised control (NOFERT) showed distinct differences in soil biochemical quality and received no N input, we calculated the correlations including (attributing it zero N-input) or excluding NOFERT (Table 4). If NOFERT was included, soil pH, C_mic_ and SOC were significantly (p < 0.05) correlated with N_2_O emissions. These parameters also showed a high level of correlation in between each other. Excluding NOFERT, total N input (N_t_ input) was most pronounced, the input of mineral N (N_min_ input) revealed a high impact on N_2_O emissions together with soil pH.

**Table 4:** Pearson correlation coefficients between cumulative area-scaled N_2_O emissions in silage maize, total N (Nt) and mineralised N (N_min_) input to maize and soil parameters sampled in the DOK trial in 2012.

	Including NOFERT					Excluding NOFERT				
	N_2_O	Nt input	N_min_ input^1^	pH	SOC	N_2_O	Nt input	N_min_ input^1^	pH	SOC
N_t_ input	0.18					0.55*				
N_min_ input^a^	0.37	0.80***				0.63**	0.72**			
pH	−0.54*	0.38	0.25			−0.63**	−0.57*	−0.33		
SOC	−0.46*	0.18	−0.04	0.67**		−0.37	−0.36	−0.44	0.54*	
C_mic_	−0.50*	0.35	0.02	0.77***	0.88***	−0.49	−0.30	−0.52*	0.57*	0.86***

Correlations were calculated across farming systems by including and excluding NOFERT.

^a^N_min_: nitrate and ammonium.

SOC – soil organic carbon, C_mic_ – microbial biomass C.

Significant levels for correlations: *p < 0.05, **p < 0.01, ***p < 0.001.

## Discussion

With this study we obtained a unique GHG flux dataset from the oldest and longest running faming system comparison trial “DOK”. This GHG monitoring campaign was started 34 years after implementation of contrasting organic and non-organic farming systems. This dataset should thus reflect system-specific physical, chemical and biological soil properties (Supplementary Table S2) resulting from long-term differentiated management of the farming systems compared.

We observed that area-scale cumulated overall N_2_O emissions were on average 40.2% lower in organic compared to non-organic farming systems. On an annual basis covering GHG sampling in silage maize and a non-leguminous green manure, organic farming emitted on average 2.78 kg less N_2_O-N ha^−1^ than non-organic farming (Table 1). These results are in accordance with the first hypothesis. The topical review demonstrated based on 70 comparisons, that organic farming emitted annually 1.05 kg less N_2_O-N ha^−1^ than non-organic farming^8^. The results from our field measurements further support the anticipated pronounced time-dependent long-term decrease of nitrous oxide emission rates^8^ as in 2013 the DOK was in the 35^th^ year while the average duration of the trials evaluated in the review was nine years. Soil quality develops on long term towards a balanced steady state of its ecosystem. The time duration since adoption of an alternative farming practice is an important issue because nitrous oxide fluxes have been shown to decrease with a pronounced time dependency^11^. Longer duration also leads to improvement in soil quality indicated e.g. by raising SOC concentration, microbial biomass and -diversity^20,21^. Thus, the finding points to an increased N_2_O mitigation potential of organic farming over time. More measurements, preferably covering complete crop rotations, are needed to confirm this observation on long-term dynamics within the context of soil quality, soil nutrient fluxes and pools.

During silage maize cropping, organic farming emitted, cumulated on area scale, on average 1.68 kg less N_2_O-N ha^−1^ compared to non-organic farming. N_2_O emissions recorded for silage maize cropping were in accordance with previous studies under similar pedoclimatic conditions: Kaiser and Ruser reported annual emissions of 2.1 kg N ha^−1^ for synthetic fertilisers and 5.3 kg N ha^−1^ for farmyard manure under a fertilisation intensity of 200 kg N ha^−1^ ^22^.

We observed pronounced N_2_O emissions after grass-clover incorporation in all farming systems as has been shown elsewhere^23^. Even in the NOFERT system pronounced N_2_O emissions were observed following grass-clover incorporation and soil preparation for maize (Fig. 1). Cumulated on area scale, N_2_O emissions for the unfertilised treatment NOFERT under silage maize were, with only 0.32 kg N_2_O-N ha^−1^, insignificantly lower than the average emissions in the fertilised non-organic treatments (Table 1). Mineral N derived from decomposition of the incorporated grass-clover ley and from soil organic matter may not have been efficiently taken up by maize plants compared with fertilised systems, as the soil in NOFERT lacks important macro nutrients such as P and K, limiting plant growth and N uptake^19^. The extreme measure of excluding fertilisation and relying primarily on soil building during grass-clover leys and N fixation of soya, as done in the NOFERT treatment, not only results in a poor yield performance and soil organic carbon losses^19,24^ resulting to soil degradation, but also leads to adverse effects in terms of GHG emissions.

Despite the pronounced difference in maize yields, yield-scaled N_2_O emissions did not differ between organic and non-organic systems in the DOK field trial (Table 2). This contrasts with the findings of Skinner *et al*., who found higher yield-scaled N_2_O emissions in organically managed soils^8^. The silage maize 2013 resulted with an average organic to non-organic yield gap of 27%. The long-standing average in the DOK, however, is only 9% for silage maize and 19% across all crops^25^. Seufert *et al*. calculated in their global meta-analysis based on 316 observations an average organic to non-organic yield ratio of 0.75 for all crops and a ratio of 0.85 for maize (74 observations)^9^, corresponding to a yield gap of 15% which is in accordance with DOK averages. The dry weather conditions during summer 2013 in Switzerland (Supplementary Fig. S1) led to generally low yields for most crops. The yield performance of organically cultivated silage maize in the DOK was apparently more impaired (−26%) than in the non-organic systems (CONMIN: −13%; CONFYM: −10%) compared to the long-term yielding average. This impairment difference might be related to the cultivar “Colisee” which was originally bred for non-organic farming and judged to be suitable for organic farming under favourable conditions. The 56% lower emissions for BIODYN compared to CONFYM can be related to the 52% lower input, but the resulting yield gap of only 27% for BIODYN (Tables 1, 2, Supplementary Table S4) indicates that this system’s N efficiency is superior and thus contributes to GHG mitigation. Our results show that organic farming management does not per se lead to higher yield-scaled N_2_O emissions.

In grass-clover, emissions were comparatively low with lowest emissions in BIODYN and CONFYM. Similar N_2_O emissions under organic and non-organic in grass-clover were found elsewhere^23,26,27^. As the GHG monitoring campaign started in the second year of the grass-clover ley, the calculation of the corresponding yield-scaled N_2_O emissions was impossible. Furthermore, yields of the 5^th^ and final cut in 2013 were rather low compared to the long-term average. The ley had to grow under very dry conditions in 2011 and could therefore never establish well. We decided against the calculation of emission factors also because mineralisation of the grass-clover ley introduced an additional source of N in all farming systems, thus questioning the explanatory power of the emission factor approach for single crops in systems with complex crop rotations which also has been concluded elsewhere^23^.

Correlation analysis (Table 4) revealed that fertiliser N input and especially the addition of mineralised N to silage maize best explained cumulative N_2_O emissions among the fertilised treatments. This is commonly observed, and a reduction of fertilising intensity is therefore suggested to be a good mitigation option^3^. The soil characteristics C_mic_, SOC and pH (Supplementary Table S2) appeared to be further explaining variables, at least when NOFERT was included in the correlation analysis. The pH was the most robust variable explaining N_2_O emissions, as its impact on N_2_O holds true with or without the inclusion of the NOFERT dataset (Table 4). Rather high N_2_O emission rates were recorded for NOFERT treatment which showed the lowest soil pH particularly during the maize and the green manure phase. The N_2_O reduction process to N_2_ may have been restricted in NOFERT soil in comparison to the other treatments. The pH dependence of the N_2_O reduction process in soil has been investigated elsewhere^28^ and has been determined recently in an accompanying laboratory experiment with soil from the DOK trial^29^. The low pH in NOFERT seems to impede functionality of the relevant N_2_O reductase enzyme in soil microorganisms^30^. In line with this, BIODYN, the farming system with the highest soil pH, showed the lowest area- and yield-scaled N_2_O emissions. We found no correlation between the pH from the starting phase in 1977 and the current N_2_O emissions. This indicates that rather management-induced changes in soil pH over time contribute to the observed changes in N_2_O emissions.

Assessing all systems including NOFERT we have further found good correlation of N_2_O emissions with SOC and microbial biomass (Table 4). It has been shown in previous studies, that the contrasting farming management induced a gradient in SOC and microbial biomass in the DOK trial, with the lowest contents in NOFERT and the highest in BIODYN^18,31^.

Studying the influence of the farming systems on microbial community structure, Esperschütz *et al*. detected distinct microbial communities in BIOORG and BIODYN compared to CONMIN and NOFERT, while samples originating from CONFYM formed a separate cluster^16^. Using a high-throughput pyrosequencing approach of bacterial and fungal ribosomal markers Hartmann *et al*. further revealed that manure addition was the most prominent factor influencing the formation of distinct bacterial and fungal communities in the different farming systems of the DOK trial^17^. However, functional gene quantification of soil microbes involved in N cycling at the end of the gas sampling campaign did not reveal substantial differences between farming systems. At best, N_2_O reducers showed effects between single systems (Supplementary Table S5). Yet it remains unclear how the shift in community composition of soil microbes in the different farming systems affects soil functionality regarding N_2_O production and reduction during cropping periods.

Cultivating soil as a habitat for soil organisms and not just as a substrate for plant growth addresses the goal of organic farming’s strive for organic matter build-up stimulating soil fertility and nutrient cycling^15^ which apparently also influences the N_2_O emission properties. Although organically managed soils rich in SOC and microbial biomass may show a higher potential for N_2_O production than those from non-organic management under denitrifying conditions^29^, this does not necessarily translate into the field situation with structured soils and system-specific fertilisation. In turn, this implies that adapted fertilisation strategy in organically managed soils prevents excessive N_2_O emissions despite higher N_2_O emission potential.

In contrast to the three comparative studies included in the meta-study of Skinner *et al*.^8^, we found no difference in area-scaled CH_4_ emissions between organic and non-organic farming. The CONFYM soils and to a lesser extent also NOFERT soils, appeared to be CH_4_ sources (Table 2). The systems BIODYN, BIOORG and CONMIN showed a pattern of CH_4_ uptake as reported for upland soils elsewhere^32–34^. It was shown that regular application of stacked cattle manure increases the biomass of methanogenic archaea^35^. This can lead to considerable CH_4_ emissions in combination with high soil water contents^35,36^. In our study, this may explain high CH_4_ emissions in CONFYM at the beginning of the silage maize vegetation phase in 2013 (Fig. 1). Manure application, ploughing and seedbed preparation during May occurred under cold and moist weather conditions (Supplementary Table S1; Supplementary Fig. S1). In contrast, application of composted organic materials where shown in an incubation experiment to transiently foster CH_4_ oxidation activity in agriculturally managed soils^37^, which could explain CH_4_ uptake in BIODYN during silage maize cropping (Table 2). Anoxic conditions due to high soil water content disable CH_4_ oxidation, which brings uptake to a standstill but allows methane from deeper layers to pass through upper soil horizons to the atmosphere^38^. This may explain the methane release in NOFERT and BIOORG during silage maize cropping (Table 2). Events with more than 30 mm rainfall or consecutive days with rain in early June increased WFPS in those systems and BIODYN to around 80%, while WFPS in CONMIN and CONFYM was distinctively lower, most probably due to the higher water uptake of their crop (Fig. 1).

This is, according to our knowledge, the first study where soil GHG flux data resulting from bioorganic and biodynamic farming systems were compared. It turned out that BIODYN showed distinctly lower area-scaled N_2_O emissions than BIOORG over the whole observation period. Even the yield-scaled N_2_O emissions were in tendency lower in BIODYN. The main management difference between BIODYN and BIOORG consists of the type of solid manure applied. This is composted farmyard manure in BIODYN and rotted farmyard manure in BIOORG, although the amount of plant available N was nearly the same for both systems (Supplementary Table S4). Composting of farmyard manure as practiced in BIODYN may raise soil pH over stacked and rotted manure application^39^. BIODYN is also the farming system with highest SOC and microbial biomass values over the years^16,18^.

Furthermore, van Groeningen *et al*. (2010) found that up to a threshold of 187 kg ha^−1^ fertiliser-N applied, corresponding to the amount applied to BIOORG, emissions remained rather stable at low flux rates to increase sharply for higher application rates^40^. They propose to focus on maximising crop N uptake together with balancing N application to crop N needs. These results are consistent to those of Shcherbak *et al*. who demonstrated an exponential increase of N_2_O emissions for fertiliser applications exceeding crop requirements^41^.

We are aware, that for a comprehensive GHG assessment of a given farming system, not only the soil-derived emissions but also all other emissions caused by the production of different (synthetic/organic) fertilisers, energy use from farm machinery and emissions caused by livestock and manure management, need to be accounted for^42^. This, however, is beyond the scope of the current study. A previous LCA study, using experimental data of the DOK trial (1985–1998), found the area-scaled (26–35%) and product-based (2–17%) energy use for farm operations such as machinery, synthetic/organic fertilisation and plant protection to be lower in the two organic systems compared to CONFYM in dependence of the fertilisation intensity^43^.

In our case, ecological intensification by the means of organic farming leads to the same yield-scaled N_2_O emissions as found for non-organic farming systems. As, compared to non-organic systems, organic farming systems were reported to deliver greater ecosystem services and social benefits in a wider context, ecological intensification would not just address climate change mitigation but, at the same time, also the other main categories of agricultural sustainability, such as socio-economic aspects^6^.

Crowder and Reganold analysed the economic performance of organic compared to conventional farming with a meta-analysis encompassing data from 55 crops on five continents over 40 years^44^. Taking current price premiums for organic products into account organic farming resulted to be with 22% to 35% more profitable and had a better benefit/cost ratio of 20% to 24% compared to non-organic. However, without price premiums organic farming resulted to be less profitable. Accounting for negative external environmental costs of agriculture, however, is likely to stimulate the expansion of organic agriculture^44^ which currently covers less than 1% of global agricultural land^45^.

## Methods

### Study site

The DOK long-term farming systems comparison trial, established 1978, is located (47°30′N; 7°32′E; 306 m asl) in Therwil, Switzerland. The soil is a Haplic Luvisol (15% sand, 70% silt and 15% clay) on deep deposits of alluvial Loess.

The trial is designed as a randomized split-block (fertilisation and crop shift), with four replicates arranged in a standard Latin square. It encompasses four farming systems – two organic ones, bio-dynamic, BIODYN (composted manure and slurry) and bioorganic, BIOORG (rotted manure and slurry); a conventional one (with manure, slurry and synthetic fertiliser) CONFYM - all of which are cultivated in a full (1.4 livestock units) as well a half-fertilised treatment. The second conventional system (exclusively synthetic fertilisation) CONMIN – has only one treatment at normal fertilisation level adjusted to soil P and K contents. An unfertilised control, NOFERT, complements the comparison. Plant protection in the organic and the unfertilised system is based on mechanical weeding, indirect disease control measures and plant extracts together with bio-controls against insects, while in the non-organic systems herbicides, fungicides and pesticides are applied^15^.

Preparation of the different manure types is performed on identical amounts of fresh manure. Stacked manure is being compacted and thus undergoes an anaerobic fermentation at about 25 to 30 °C. Rotted manure is being shifted once after four weeks, while compost is turned on a weekly basis. Losses of dry matter, organic matter and nitrogen during preparation account to 22, 29 and 19% for stacked manure, 32, 47 and 30% for rotted manure and 42, 57 and 33% for composted manure^46^.

The seven-year crop rotation is identical for all farming systems and is cultivated in three rotation units with a time shift of one year. The 6^th^ crop rotation since the onset of the trial started 2013. The rotation encompasses silage maize followed by green manure of a *Brassica chinensis x Brassica rapa* breed over winter (first year), soybean (second year), winter wheat-1 and green manure (third year), potatoes (fourth year), winter wheat-2 (fourth and fifth year) and two years of a grass-clover ley (years 6 and 7)^15^.

The local climate is favourable for agriculture being rather mild and dry with annual (1981–2010) means of 10.5 °C and 842 mm rainfall (1961–1990: 9.6 °C, 778 mm) recorded at Basel/Binningen (BAS; WMO: 06601; 7°35′E; 47°32′N; 316 m asl) by the Federal Office for Meteorology and Climatology.

### Monitoring period

CONMIN and the fully fertilised treatments of the farming systems BIODYN, BIOORG and CONFYM were chosen for investigation as they are representative for common agricultural practices in Switzerland. NOFERT was included as a control in the greenhouse gas measurement campaign at a later stage before ley termination in March 2013. Starting on 24 Aug 2012, GHG flux recordings, concomitant soil and agronomic analyses covered a sequence of grass-clover (256 days) – silage maize (140 days) – green manure (175 days) ending on 18 March 2014. Further details and farming operations are displayed in Supplementary Table S1, amount of fertiliser applied to grass-clover in Supplementary Table S3, and fertiliser rates for silage maize in Supplementary Table S4. Yields for silage maize are displayed in Table 3 together with yield-scaled N_2_O emissions.

Supplementary Fig. S1 displays monthly means for temperature, rainfall and sunshine hours related to the norm period of 1980–2010. In 2012, the mean annual temperature was 10.9 °C and rainfall amounted to 1048 mm in the Basel area. During the first half of November 2012, extreme rainfall events led to an annual surplus of over a third of normal. In 2013, the mean annual temperature was 10.3 °C and rainfall amounted to 908 mm. The winter was unusually cold and prolonged until end of February. Spring was extremely cloudy, rainy and cool. With only half of the mean hours of sunshine it turned out to be the least sunny spring since 1833. Rainfall amounted to roughly 140% of normal. At the end of May 2013, the weather turned nearly to the other extreme with much sunshine, occasionally hot, so that a lasting monthly temperature surplus compared to normal resulted until the end of October. Winter 2013/2014 was rather warm, nearly frost-free.

### Greenhouse gas sampling

Soil gas fluxes of N_2_O, CH_4_ and CO_2_ were sampled with manual closed chambers of 30 cm inner diameter and 0.7 L volume as described elsewhere^47,48^. Soil collars were removed for tillage operations only. Chamber height was adjusted to the growing crop with tubular interfaces. Gas flux samplings were performed on a weekly base with additional samplings covering management operations. This extended manual sampling scheme has been shown to provide less than 10% deviation compared to near-continuous automated gas measurements^49^. Samplings were restricted to between nine in the morning and noon (apparent solar time) when actual trace gas fluxes best represent the daily mean of the highly variable flux rates^50^. One gas sampling consisted of four gas samples taken within 50 to 90 minutes with 20 ml plastic syringes. Gas samples were transferred into evacuated 12 ml vials creating an overpressure in vials which was needed to allow analysis via gas chromatography with an autosampler. Gas analysis involved HayeSep Q 80/100 columns at 100 °C oven temperature and N_2_O detection with an electron capture detector (µECD), CO_2_ reduction with a methanizer and subsequent detection of CO_2_ and CH_4_ with a flame ionisation detector (FID). Three calibration gases with concentrations from ambient to 10 times ambient were used. Fluxes were calculated with robust linear and non-linear models of the HMR function (package HMR) in R (R Core team) which was slightly adjusted as described elsewhere^51^. Cumulative gas emissions were integrated over time using the trapezoidal rule.

### Soil sampling

Every gas sampling included a soil sampling (0–20 cm) bulked per treatment to determine soil water content and mineral soil nitrogen (N_min_). Soil water content (WC) was determined gravimetrically with 24 h drying at 105 °C. Water filled pore space (WFPS) was calculated based on corresponding bulk densities. Soil extractions with 0.01 M CaCl_2_-solution (1:4 w/v) were subject to 45 minutes of shaking and filtration before spectrophotometric analysis was employed to determine nitrite, nitrate and ammonium concentrations via continuous flow analysis. A basic sampling for microbial biomass, soil organic carbon and further variables was performed every year during spring time, shortly before starting the farming operations. Determination of soil pH was performed with a soil-water suspension, soil organic carbon concentration by wet oxidation (Walkley Black) and microbial biomass by chloroform fumigation extraction using 0.5 M K_2_SO_4_ (1:4 w/v) as described elsewhere^18^.

### Statistical analysis

All calculations were accomplished in R software^52^. Field replicates of gas samples were pooled per plot for statistical analysis. Cumulated N_2_O emissions and soil data of 2012 were assessed with a linear mixed effect model with field replication as random effect and clay content as covariate. Relations of cumulated N_2_O emissions in maize with N input and soil data were calculated with the Pearson rank correlation (Hmisc package). Time series correlation of N_2_O fluxes that were pooled per treatment, with ancillary soil data (n = 1 per treatment) was calculated with a linear mixed effect model with farming system treatments as random effect and sampling date per treatment as correlation term (repeated measurements, nlme package). Due to non-normal distribution, cumulated CH_4_ fluxes were assessed with a Kruskal Wallis test for treatment and farming system differences (PCMCR package).

## Supplementary information

Supplementary Information

## Data Availability

The datasets generated during and/or analysed during the current study are available from the corresponding author on request.
