# Supplementary material for: The impact of long-term organic farming on soil-derived greenhouse gas emissions
Source: Sci Rep. 2019 Feb 8;9:1702. doi: 10.1038/s41598-018-38207-w (PMC6368562; doi:10.1038/s41598-018-38207-w)
Supplement: Supplementary file 1 — Supplementary Information [file 41598_2018_38207_MOESM1_ESM.pdf]

Title: The impact of long-term organic farming on soil-derived greenhouse gas emissions

Authors: Colin Skinner[a], Andreas Gattinger[a,b]*, Maike Krauss[a], Hans-Martin Krause[a], Jochen Mayer[c], Marcel G.A. van der Heijden[d,e,f], Paul Mäder[a]

Supplementary Information

Table S1: Farming operations accomplished during the grass-clover – silage maize –green manure crop sequence from 24 August 2012 to 18 March 2014.

ID	Date	Farming System	Farming operation
1	*24 August 2012*		*First sampling of GHG fluxes*
2	27 August 2012	All farming systems	4th cut of grass-clover in 2012
3	8 October 2012	All farming systems	5th cut of grass-clover in 2012
4	8 May 2013	All farming systems	Last cut of grass-clover before termination
5	13 May 2013	CONFYM	Solid manure application*
6	14 May 2013	All farming systems	Ploughing and rolling
7	16 May 2013	BIODYN, BIOORG	Solid manure application*
8	27 May 2013	CONMIN	Mineral fertilization (CAN, TSP, PK, K)
8	27 May 2013	All farming systems	Manure incorporation (Rotary harrow)
9	28 May 2013	All farming systems	Seeding of silage maize (cv. 'Colisee')
10	14 June 2013	BIODYN, BIOORG	Mechanical weeding (Flex-tine weeder)
11	17 June 2013	BIODYN, CONFYM, BIOORG	Liquid manure (= slurry) application
12	20 June 2013	BIODYN, BIOORG	Mechanical weeding (Cultivator)
13	26 June 2013	CONFYM, CONMIN	Herbicide application
14	2 July 2013	CONFYM, CONMIN	Mineral fertilization (CAN)
14	2 July 2013	BIODYN, BIOORG	Liquid manure (= slurry) application
15	9 July 2013	CONFYM, CONMIN	Mineral fertilization (CAN)
15	9 July 2013	BIODYN, BIOORG	Mechanical weeding (Cultivator)
16	17 July 2013	BIODYN, BIOORG	Manual weeding in rows
17	25 September 2013	All farming systems	Silage maize harvest
18	8 October 2013	All farming systems	Mulching maize stubbles
19	9 October 2013	All farming systems	Seeding of green manure<br>(Brassica chinensis x Brassica rapa)
20	*18 March 2014*		*Last sampling of GHG fluxes*

ID numbers refer to corresponding farm operations given in Figure 1.

Mineral fertilizers: CAN – calcium ammonium nitrate, TSP – triple super phosphate, PK - Patentkali®, Kali®

*: Application of solid manure before or after ploughing depends on respective system specific fertilization strategy

Table S2. Means and standard errors (SE) of soil pH, soil organic carbon (SOC) and microbial biomass C (C_{mic}) concentrations from 2012.

	pH (H_2O)			SOC (g kg^{-1})			C_{mic} (mg kg^{-1})		
Farming System	Mean	SE		Mean	SE		Mean	SE	
BIODYN	6.70	0.03	a	13.97	0.52	a	486.9	32.7	a
BIOORG	6.36	0.03	b	11.80	0.44	a	368.7	8.1	b
CONFYM	6.35	0.08	b	11.33	0.38	ab	369.4	5.9	b
CONMIN	6.53	0.08	b	11.38	1.43	bc	331.7	34.0	c
NOFERT	5.93	0.17	c	9.80	1.03	c	248.8	26.4	d
ANCOVA	F-value	p-value		F-value	p-value		F-value	p-value	
(Intercept)	16839.0	<0.0001		4138.6	<0.0001		2104.0	<0.0001	
Clay content	2.32	0.155		45.5	<0.0001		18.0	0.001	
Farming System	12.78	<0.0001		15.8	<0.0001		66.3	<0.0001	
Contrasts	t-value	p-value		t-value	p-value		t-value	p-value	
org vs. non-org	1.15	0.246		3.72	<0.0001		7.06	<0.0001	

ANCOVA per farming system (n = 4). Letters = Post-hoc Tukey test (p<0.05).
Contrasts: Post-hoc t-test on pairwise comparisons of org (BIODYN + BIOORG) and non-org (CONFYM + CONMIN) systems (n = 8)

Table S3. Amount of organic and mineral fertiliser applied to 2nd year grass-clover in 2012.

Farming System	Fertilizer type	Amount [ha^{-1}]	Nt [kg ha^{-1}]	N_{min}[1] [kg ha^{-1}]	OM (DM) [kg ha^{-1}]
BIODYN	Slurry	80 m^3	74	39.8	1266
BIOORG	Slurry	50 m^3	48	29.1	443
CONFYM	Slurry	140 m^3	212	164	1489
CONMIN	Mineral fertilizer	700 kg	140	140	0
NOFERT	none				

[1]: N_{min}: nitrate and ammonium

Organic matter (OM) expressed in dry matter (DM)

Table S4. Amount of organic and mineral fertiliser applied to silage maize in 2013.

Farming System	Fertilizer type	Amount [ha^{-1}]	N_t [kg ha^{-1}]	N_{min}[1] [kg ha^{-1}]	OM (DM) [kg ha^{-1}]
BIODYN	Composted manure	20 Mg	98	6	2932
	Slurry	60 m^3	41	24	675
	Sum		139	30	3607
BIOORG	Rotted manure	20 Mg	136	9	3670
	Slurry	60 m^3	46	28	486
	Sum		182	37	4156
CONFYM	Stacked manure	35 Mg	167	43	5303
	Slurry	40 m^3	58	49	344
	Mineral fertilizer	550 kg	110	110	0
	Sum		335	202	5647
CONMIN	Mineral fertilizer	850 kg	170	170	0
NOFERT	none				

[1:] N_{min}: nitrate and ammonium

Organic matter (OM) expressed in dry matter (DM)

Table S5. Functional gene abundance for nitrification (bacterial and archaeal *amoA*), denitrification (*nirS, nirK*) and N_2O reduction (*nosZ, nosZ-II*) in two organic- (BIODYN, BIOORG) and two conventional farming systems (CONFYM, CONMIN) compared to an unfertilised control (NOFERT). Composite samples were taken from each parcel to a depth of 10 cm at 18 March 2014. Data show mean (n=4) and standard errors. Details of functional gene quantification are described elsewhere (28).

	archaeal amoA			*bacterial amoA*			*nirS*		
Farming System	Mean	SE		Mean	SE		Mean	SE	
BIODYN	3.62E+07	6.15E+06	a	2.82E+05	4.09E+04	a	6.58E+07	9.78E+06	a
BIOORG	3.79E+07	7.17E+06	a	3.63E+05	1.36E+05	a	4.99E+07	5.65E+06	a
CONFYM	3.63E+07	6.14E+06	a	6.33E+05	2.12E+05	a	6.35E+07	1.07E+07	a
CONMIN	3.78E+07	7.60E+06	a	6.57E+05	3.10E+05	a	6.47E+07	1.69E+07	a
NOFERT	3.41E+07	7.28E+06	a	1.45E+05	5.24E+04	a	4.92E+07	1.31E+07	a
ANCOVA	F-value	p-value		F-value	p-value		F-value	p-value	
(Intercept)	32.48	<0.0001		20.80	0.001		65.88	<0.0001	
Clay content	0.28	0.608		0.87	0.371		2.13	0.172	
Farming System	0.79	0.556		2.22	0.134		0.76	0.575	
Contrasts	t-value	p-value		t-value	p-value		t-value	p-value	
org vs. non-org	0.01	0.989		-1.98	0.048		-0.65	0.519	

	nirK			*nosZ*			*nosZ-II*		
Farming System	Mean	SE		Mean	SE		Mean	SE	
BIODYN	1.31E+08	2.39E+07	a	6.45E+07	5.98E+06	b	5.18E+08	6.08E+07	a
BIOORG	1.84E+08	1.84E+07	a	9.84E+07	8.23E+06	a	4.33E+08	4.46E+07	ab
CONFYM	1.57E+08	2.30E+07	a	7.40E+07	8.54E+06	ab	4.26E+08	3.57E+07	ab
CONMIN	1.79E+08	3.52E+07	a	7.20E+07	6.29E+06	ab	4.42E+08	6.12E+07	ab
NOFERT	1.48E+08	2.45E+07	a	6.66E+07	8.51E+06	b	3.26E+08	5.87E+07	b
ANCOVA	F-value	p-value		F-value	p-value		F-value	p-value	
(Intercept)	224.66	<0.0001		456.76	<0.0001		480.43	0.000	
Clay content	1.79	0.208		0.87	0.371		6.57	0.026	
Farming System	1.24	0.350		2.78	0.081		2.84	0.077	
Contrasts	t-value	p-value		t-value	p-value		t-value	p-value	
org vs. non-org	-0.44	0.660		1.07	0.285		0.92	0.357	

ANCOVA per farming system (n = 4). Letters = Post-hoc Tukey test (p<0.05).
Contrasts: Post-hoc t-test on pairwise comparisons of org (BIODYN + BIOORG) and non-org (CONFYM + CONMIN) systems (n = 8)

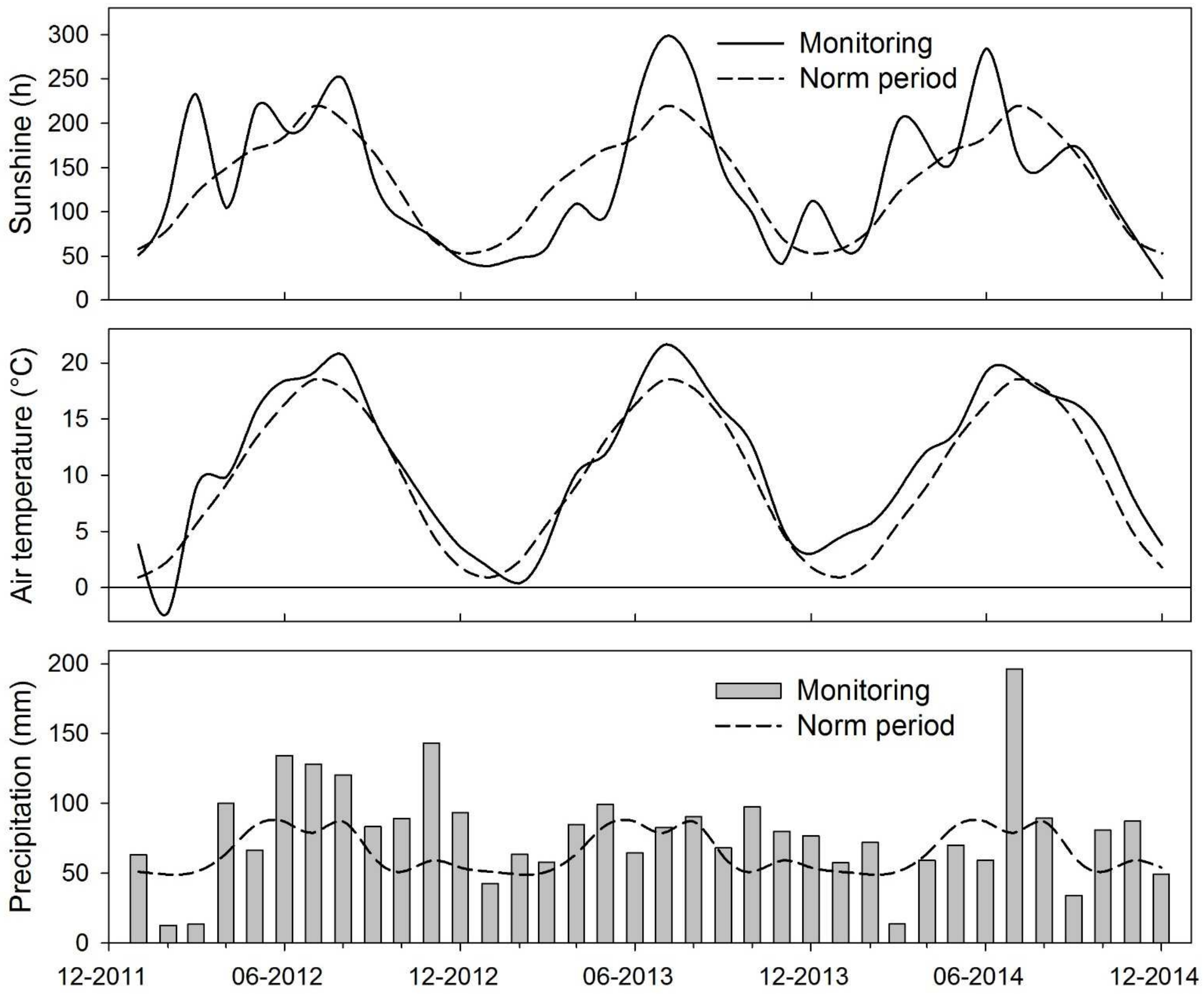

Figure S1: Comparison of mean sunshine hours, air temperature and precipitation during 2012 to 2014 with the norm period 1981 to 2010 values.
